# On the Death Wound of General Abercromby

**Published:** 1804-08-01

**Authors:** 


### *To the Editors of the Medical and Physical Journal.*

GENTLEMEN,

WAR and disease are the concomitants of our existence. Human nature seems pregnant with the seeds of these evils even on its birth. Both oftentimes assume an almost unlimited extension in their devastating powers, by which all descriptions are precipitated into Eternity. The miscellany of human suffering galls even the pen of traditional fidelity, and often deters the mind's retrospect into into the complication of misery—the direful consequences of war and disease. But the historian must not stop here, every event is incumbent on him to relate; the concurrence of contingencies, the unique influence or the complex issue from the co-operation of efficient principles, and the eventful explosion of preconcerted plans, form the field for his historical powers. The faithful delineation of a campaign challenges at once all the warm emotions of the breast; a patriotic enthusiasm is excited at the recital of native valour, and the ardent glow of sympathy, cherished by the tale of extraordinary virtue and valour, follows with gratitude the victor to his grave.—What breast was not animated with every worthy feeling on the catastrophe of our brave Abercromby; and although misfortune deprived the field of our General, he will ever continue to live in the memory of a grateful posterity.—It is time that a true and accurate history of this great man's misfortune should be given to the world, and I therefore, through the medium of your invaluable Journal, call upon that gentleman (Mr. Gillham*), whom that deserving and meritorious artist† has exhibited as the medical officer and operator on the occasion. There have been so many different accounts given to the public, and none of which being clearly and professionally detailed, that I embrace this period, although distant, to request of that medical gentleman, whom the public now recognizes‡ as the surgical attendant, to describe the precise place, depth, superficial extent, and external consequences or termination of the wound which occasioned the loss of the virtuous General. As I am confident that most medical men, and more especially those who served during the campaign in Egypt, are desirous for a faithful history of the case, and as Mr. Gillham has not given to the public the least information upon this important and interesting subject, I have not the least hesitation, from my personal knowledge of Mr. G. to assert, that the medical part of the expedition will be soon gratified by perusing the interesting particulars of the fatal case. I am, &c.

*Chancery Lane, July* 12, 1804. AN OLD CAMPAIGNER.

---

* Mr. Gillham having received great and irreparable injury in his eyes from the powerful influence of the climate, has been placed on the invalid list, and now practices as a surgeon and apothecary in this metropolis or its environs.

† Mr. Porter, one of the most ingenious and meritorious artists of the present epoch.

‡ The picture of the death of Sir R. Abercromby, by Mr. Porter.

